# Applications of Microencapsulated *Bifidobacterium Longum* with *Eleutherine Americana* in Fresh Milk Tofu and Pineapple Juice

**DOI:** 10.3390/nu7042469

**Published:** 2015-04-03

**Authors:** Atchara N. Phoem, Suphitchaya Chanthachum, Supayang P. Voravuthikunchai

**Affiliations:** 1 Department of Biology and Applied Biology, Faculty of Science and Technology, Songkhla Rajabhat University, Muang, Songkhla 90000, Thailand; E-Mail: aphoem@yahoo.com; 2 Department of Food Technology, Faculty of Agro-industry, Prince of Songkla University, Hat Yai, Songkhla 90112, Thailand; E-Mail: suphitchaya.c@psu.ac.th; 3 Department of Microbiology and Excellent Research Laboratory on Natural Products, Faculty of Science and Natural Product Research Center of Excellence, Prince of Songkla University, Hat Yai, Songkhla 90112, Thailand

**Keywords:** *Bifidobacterium longum*, *Eleutherine americana*, fresh milk tofu, microencapsulation, pineapple juice

## Abstract

*Bifidobacterium longum* was microencapsulated by extrusion technique and added in fresh milk tofu and pineapple juice. Microencapsulation of *B. longum* with *Eleutherine americana* extract, oligosaccharides extract, and commercial fructo-oligosaccharides was assessed for the bacterial survival after sequential exposure to simulated gastric and intestinal juices, and refrigeration storage. Microencapsulated *B. longum* with the extract and oligosaccharides extract in the food products showed better survival than free cells under adverse conditions. Sensory analysis demonstrated that the products containing co-encapsulated bacterial cells were more acceptable by consumers than free cells. Pineapple juice prepared with co-encapsulated cells had lower values for over acidification, compared with the juice with free cells added. This work suggested that microencapsulated *B. longum* with *E. americana* could enhance functional properties of fresh milk tofu and pineapple juice.

## 1. Introduction

Functional foods provide a health benefit that goes beyond general nutritional content, and particularly foods containing probiotics are a natural way of enhancing the functionality of food products [1]. Viability of probiotics must be maintained throughout the product’s shelf-life and gastrointestinal conditions. Maintenance of their survival until they reach the gastrointestinal tract is one of the key requirements for health benefit. International Dairy Federation (IDF) has suggested that a minimum of 10^7^ CFU probiotic bacterial cells should be alive at the time of consumption per gram of the product. Bifidobacteria, a group of commonly selected probiotics, are dominant in infant gut microbiota [2].

The most common probiotic-containing foods are dairy products. The dairy products have a place in delivering probiotics to human gut, as they provide a suitable environment for their viability [2]. However, probiotics may show low survival in fermented milk products due to their acidic nature. Probiotics may be incorporated into fresh milk tofu which is a suitable environment for probiotic delivery in human foods. The fresh milk tofu shows pH of 6.2 and contains more solid content, which creates the possibility for satisfactory viability of probiotics. Lactose intolerance, cholesterol content, and allergic milk proteins are not suitable for intake of dairy products and give rise to the development of non-dairy products as an ideal delivery media for probiotics [3]. Fruit juices, particularly pineapple juice, could be a healthy probiotic carrier food due to its high nutritional value including vitamins and antioxidants [4]. Moreover, there is no starter culture in the juice like yoghurt which can compete with probiotics, and loss in viability occurs during refrigeration storage [5].

Microencapsulation by extrusion technique has been applied for the protection of the probiotics against gastrointestinal transit and food products. A combination of alginate with prebiotic oligosaccharides produces beads with a good integrate structure resulted in the improvement of probiotic viability in adverse environmental conditions [6]. It has previously been reported that microencapsulation of probiotics with resistant starch [7] and chitosan [8] was able to increase the survival of probiotics in dairy and non-dairy products during refrigeration storage and gastrointestinal conditions.

Prebiotics oligosaccharides appear naturally in fruits, vegetables, milk, and honey. *Eleutherine americana* Merr. is a herbal plant whose red bulb has been used in Asian cuisine. There have been a number of studies on *E. americana* extract for applications in clinical [9] and food preservation [10]. Furthermore, hot water extract from *E. americana* has been used to provide growth stimulation on beneficial bacteria [11]. However, survival of microencapsulated probiotic with *E. americana* oligosaccharides in fresh milk tofu and pineapple juice has not yet been documented. Furthermore, no reports were found in the literature about the sensory evaluation of each product.

Therefore, the objectives of this study were to evaluate the enhanced survival of microencapsulated *Bifidobacterium longum* with *Eleutherine americana* extract and oligosaccharides extract in food products under gastrointestinal conditions and refrigeration storage. Sensory scores of the food products were assessed.

## 2. Materials and Methods

### 2.1. Probiotic Bacteria from Infant Faeces

Faeces from healthy infants were collected and bifidobacteria were isolated from collected samples by the method of Phoem and Voravuthikunchai [12]. Twenty-three isolates were investigated for characterization as potential probiotics. *Bifidobacterium longum* (isolate 4) showed good probiotic properties including high acid tolerance at pH of 2 and bile resistance at 0.30% oxgall, high protein, lipid, starch, *Eleutherine americana* extract utilizations, good antibacterial activity against *Staphylococcus aureus* ATCC 27664 and *Salmonella* Typhimurium ATCC 13311. *B. longum* was used as the target strain in this present work.

*B. longum* cells were cultivated in 50 mL of MRS broth (Merck, Damstadt, Germany) supplemented with 0.05% (w/v) l-cysteine hydrochloride and incubated at 37 °C under anaerobic condition for 24 h. They were centrifuged at 10,000 g for 10 min, at 4 *°*C. The pellets were washed with 0.1% (w/v) pre-reduced normal saline solution and re-suspended in 10 mL of 0.1% (w/v) pre-reduced peptone solution. The cell suspensions were adjusted to final concentration of approximately 1 × 10^10^ CFU mL^−^^1^. They were divided into two groups: one part was used for microencapsulation and the other as free cells.

### 2.2. Eleutherine Americana Extract and Oligosaccharides Extract

Bulbs of *E. americana* were collected from Songkhla, Thailand. They were extracted by hot water according to the method of Phoem and Voravuthikunchai [11]. Briefly, the bulbs were extracted with distilled water in ratio of 1:10 (w/v) at 80 *°*C for 1 h. The filtrates were dried by freeze dryer (Flexi Dry, Germany).

*E. americana* extract was partially-purified using *Saccharomyces cerevisiae* BCC 12652 and then precipitated twice by 80% ethanol at 4 °C for 12 h [11]. The oligosaccharides extract was analyzed for fructo-oligosaccharides by High Performance Liquid Chromatography (HPLC 1100, Hewlette Packard, Germany). Commercial fructo-oligosaccharides (Sigma-Aldrich, Steinheim, Germany) was used as reference. The extract, oligosaccharides extract, and commercial fructo-oligosaccharides were dissolved in sterile distilled water and used for further studies.

### 2.3. Microencapsulation of Bifidobacterium Longum with Eleutherine Americana

The extrusion technique was performed for microencapsulation process as described previously [13]. Briefly, two milliliters of cell suspension (1 × 10^10^ CFU mL^−^^1^) were mixed with 16 mL of sterile 2% (w/v) sodium alginate solution (Fluka, Switzerland). Two milliliters of *E. americana* extract, oligosaccharides extract, and commercial fructo-oligosaccharides were separately added to the above mixtures to make final concentration of 1% (w/v). Final concentration of cell suspension in the mixture was about 1 × 10^9^ CFU mL^−^^1^. Then, it was injected through a syringe needle size 23G (Nipro, Japan) into sterilized 0.1 M CaCl_2_ solution (Difco, Dickinson, TX, USA) from the distance of 10 cm that formed beads. Beads were allowed to harden for 30 min in CaCl_2_ solution. They were washed twice with 0.85% (w/v) pre-reduced normal saline solution and stored in 0.1% (w/v) pre-reduced peptone solution (pH 6) at 4 °C until use. The free cells were used as control.

### 2.4. Application of Microencapsulated Bifidobacterium Longum in Fresh Milk Tofu (Dairy Product)

#### 2.4.1. Preparation of Fresh Milk Tofu

The ingredients for fresh milk tofu were 7 g of agar (Pearl Mermaid, Bangkok, Thailand), 50 g of sugar, 700 mL of Ultra-High-Temperature plain milk (UHT, Nongpho, Ratchaburi, Thailand), and 300 mL of water. Agar was dissolved in water and stirred until boil. Sugar and UHT plain milk were added with high-speed stirring. Heating was continued to 65 °C and the mixture was kept at this temperature for 15–20 min. The mixture was divided into four equal fractions and cooled to 45 °C. Microencapsulated *B. longum* with *E. americana* extract, oligosaccharides extract, commercial fructo-oligosaccharides, and free cells were added aseptically into the mixture. Beads and free cells were added at concentrations about 1 × 10^9^ CFU g^−^^1^ and 1 × 10^9^ CFU mL^−^^1^, respectively. The ratio of beads and free cells to fresh milk tofu was 1:10. The fresh milk tofu (pH 6.2) was distributed in sterile plastic cups, topped with UHT plain milk plus sugar, and packed with sterile plastic lids. All experiments were performed in duplicate.

#### 2.4.2. Survival of Microencapsulated *Bifidobacterium Longum* after Sequential Incubation in Simulated Human Gastric and Intestinal Juices

Microencapsulated *B. longum* and free cells were stored in fresh milk tofu at 4 °C for 0, 2, 4, and 6 days and determined for their survival after exposure to simulated gastric and intestinal juices. Briefly, simulated gastric juice was mimicked by hydrochloric acid (HCl) buffer of pH 2 containing: NaCl-8 g L^−^^1^; KCl-0.2 g L^−^^1^; Na_2_HPO_4_·2H_2_O-8.25 g L^−^^1^; NaH_2_PO_4_-14.35 g L^−^^1^; CaCl_2_·2H_2_O-0.1 g L^−^^1^; MgCl_2_·6H_2_O-0.18 g L^−^^1^ and pepsin (Sigma-Aldrich)-3 g L^−^^1^ as described by Sandoval-Castilla *et al.* [14]. The fresh milk tofu containing microencapsulated *B. longum* (10 g) and free cells (10 mL) were added to 90 mL of simulated gastric juice and incubated at 37 °C under anaerobic condition for 3 h in a rotary shaker. One gram of fresh milk tofu containing microencapsulated *B. longum* and free cells (1 mL) was added to 9 mL of 0.1 M pre-reduced phosphate buffer (pH 7.4) followed by homogenization in stomacher for 5 min. The samples were then centrifuged at 10,000 *g* for 10 min, at 4 °C. The pellet was serially diluted with normal saline solution and plated on MRS agar modified with the addition of 0.05% (w/v) l-cysteine hydrochloride. The plates were incubated at 37 °C under anaerobic condition for 48 h. After incubation, viable cells were counted and expressed as log colony-forming units per gram (log_10_ CFU g^−^^1^). The samples after having been exposed to simulated gastric juice were centrifuged, washed with 0.85% (w/v) pre-reduced normal saline solution, and deposited in 9 mL of simulated intestinal juice (pH 7.4) containing: NaCl-6.50 g L^−^^1^; KCl-0.84 g L^−^^1^; CaCl_2_-0.22 g L^−^^1^; NaHCO_3_-1.39 g L^−^^1^; *L*-cysteine hydrochloride-0.50 g L^−^^1^; bile salt (Difco)-3 g L^−^^1^; and pancreatin-1 g L^−^^1^ (Sigma-Aldrich). They were anaerobically incubated at 37 °C for 3 h in a rotary shaker. The samples were centrifuged, washed with normal saline solution, and counted of viable cells as described in Section 2.4.2. All experiments were performed in duplicate.

#### 2.4.3. Survival of Microencapsulated *Bifidobacterium Longum* under Refrigeration Storage

The effect of refrigeration storage at 4 °C for 0, 2, 4, and 6 days in the survival of microencapsulated *B. longum* and free cells was assessed by following a modified method of Brinques and Ayub [15]. They were used for viable cell count after depolymerization of their cells in stomacher as described in Section 2.4.2. All experiments were performed in duplicate.

#### 2.4.4. Sensory Evaluation

Sensory evaluation of stored fresh milk tofu was carried out according to the method proposed by Bergara-Almeida *et al.* [16]. The study has been approved by the Ethics Committee of Prince of Songkla University, Thailand. A total of 20 trained panelists evaluated the samples for attributes of appearance, colour, texture, taste, and overall acceptability at 4 °C for 0, 2, 4, and 6 days. All experiments were performed in duplicate. Sensory evaluation was based on a nine-point hedonic scale, where 9 = extremely desirable, 5 = moderately desirable, and 1 = extremely undesirable. Five samples of each food product containing co-encapsulated *B. longum* with *E. americana* extract, oligosaccharides extract, and commercial fructo-oligosaccharides, non-encapsulated *B. longum*, and control (without *B. longum* and co-encapsulating agents), were presented to the panelists in individual plastic coded containers. The samples were randomly presented to the panel group at each session. Water was used for mouth rinsing between samples.

### 2.5. Application of Microencapsulated Bifidobacterium Longum in Pineapple Juice (Non-Dairy Product)

#### 2.5.1. Preparation of Pineapple Juice

Fresh raw pineapples (*Ananas comosus* (Linn.) Merr.) were obtained from a local market and washed in water. The fruit was peeled, cut, and ground with high speed for 1 min (Tefal Kaleo, France). The sample was passed through a mesh screen to remove pieces of flesh. One liter of the sample was sterilized and cooled to 25 °C. Microencapsulated *B. longum* with *E. americana* extract, oligosaccharides extract, commercial fructo-oligosaccharides, and free cells were separately added aseptically into pineapple juice. Beads and free cells were added at concentrations about 1 × 10^9^ CFU g^−^^1^ and 1 × 10^9^ CFU mL^−^^1^, respectively. The ratio of beads and free cells to the juice was 1:10. The pineapple juice (pH 3.8) was allocated to sterile bottles, and packed with sterile plastic lids. All experiments were done in duplicate.

#### 2.5.2. Survival of Microencapsulated *Bifidobacterium Longum* after Sequential Incubation in Simulated Human Gastric and Intestinal Juices

Microencapsulated *B. longum* and free cells were kept in pineapple juice at 4 °C for 0, 15, 30, and 45 days and their survival was determined as the similar method described in Section 2.4.2.

#### 2.5.3. Survival of Microencapsulated *Bifidobacterium Longum* under Refrigeration Storage

Pineapple juice containing microencapsulated *B. longum* and free cells were performed in the similar method as described in Section 2.4.3 and their survival was determined by the day of 0, 15, 30, and 45.

#### 2.5.4. Sensory Evaluation

Sensory evaluation of refrigerated pineapple juice during 45 days was treated in a similar way as described in Section 2.4.4.

#### 2.5.5. Production of Organic Acids in Pineapple Juice

Production of acetic and lactic acids in pineapple juice stored at 4 °C for 0, 15, 30, and 45 days was determined by modified the method of Holdeman *et al.* [17]. The samples were centrifuges at 10,000 *g* for 15 min, at 4 °C and supernatant were estimated by gas chromatography equipped with a flame ionization detection and HP-INNOWAX capillary column. The concentration of the acids was quantified by comparing with peak area of respective standard. The pH of the samples was determined with a pH meter (Beckman Instruments, Fullerton, CA, USA). All experiments were done in duplicate.

### 2.6. Statistical Analysis

The data were reported as mean ± standard deviation (S.D.). Differences among groups were examined for statistical significance by analysis of variance (ANOVA). The criterion for significance was *p* *<* 0.05.

## 3. Results and Discussion

### 3.1. Viability of Microencapsulated Bifidobacterium Longum in Fresh Milk Tofu

The diameter of beads made by the extrusion technique was 1.65–2.05 mm and their shapes were round. The type of co-encapsulating agents had no influence on the size of the beads. Survival of microencapsulated *B. longum* with extracts and free cells in fresh milk tofu after sequential incubation in simulated gastric and intestinal juices is shown in Table 1. The initial number of *B. longum* cells were in the range of 9.30–9.46 log_10_ CFU g^−^^1^ beads. The beads made by extrusion technique were kept at 4 °C for 0, 2, 4, and 6 days. The number of *B. longum* cells reduced after refrigeration storage. Survival rates of co-encapsulated cells before and after exposure to acidic and enzymatic conditions were better than those of non-encapsulated cells (*p <* 0.05). The alginate used comprised high mannuronic acid (M) and guluronic acid (G). M:G ratio can be manipulated to increase the permeability of gels to enhance the survival of co-encapsulated cells [8]. In addition, microencapsulated *B. longum* with *E. americana* might be acting as buffer against acids. The presence of food may affect survival as the probiotics may not be exposed to acid and enzyme [18]. The number of microencapsulated cells decreased when exposed to the simulated juices at day 4 and 6. The highest survival of viable cells resulted from microencapsulation with the oligosaccharides extract at day 6. Higher enzymatic resistance of the oligosaccharides extract might be due to presence of β and α-glycosidic linkage in the oligosaccharides extract. The viability of *B. longum* microencapsulation with the oligosaccharides extract decreased from 9.24–8.23 log_10_ CFU g^−^^1^ after exposure to the simulated juices.

Effect of refrigeration storage on viability of microencapsulated B. longum with extracts and free cells is shown in Table 1. Initial cell concentration of B. longum was in the range of 9.44–9.54 log_10_ CFU g^−^^1^ beads. The cell numbers of B. longum reduced after refrigeration storage. The viability of co-encapsulated cells was higher than that of non-encapsulated cells at day 2, 4, and 6 (p < 0.05). There was a reduction of pore size, thus hindering the interaction between cells and the composition of food product during storage [18]. The highest numbers of B. longum were observed when microencapsulated with oligosaccharides extract on day 6. The survival of microencapsulated *B. longum* with the oligosaccharides extract was 9.24 log_10_ CFU g^−1^. Accordingly, microencapsulated probiotics with prebiotics improved the viability than that of free cells in cheese [7,19], ice cream [20], and frozen yoghurt [21] during refrigeration storage.

### 3.2. Sensory Analysis of Fresh Milk Tofu

Sensory scores of fresh milk tofu containing microencapsulated *B. longum* with co-encapsulating agents, free cells, and control at 0, 2, 4, and 6 days of refrigeration storage are presented in Table 2. Initially, there was no significant difference in appearance, colour, texture, taste, and overall acceptability in all samples (*p >* 0.05). Higher score of fresh milk tofu containing microencapsulated *B. longum* was maintained during refrigeration storage whereas the scores of free cells and control decreased after storage period. Overall acceptability of fresh milk tofu samples containing co-encapsulated cells was higher than the samples with free cells at day 4 and 6 (*p <* 0.05). The type of co-encapsulating agents had no influence on sensory scores of all samples. The mean scores for fresh milk tofu containing microencapsulated *B. longum* ranged between 7.26 and 7.56 (where 7 = like moderately) for overall acceptability. The use of prebiotics as a co-encapsulating agent could have influenced smoothness of the food samples. Casein rearrangement around the microcapsules could result in continuous structure. In addition, co-encapsulating agents used in extrusion technique may have influenced the grittiness of the samples [22]. On the other hand, this could have been the reason that microencapsulated *B. longum* with *E. americana* appeared white and did not have an impact on color. Accordingly, sensory scores of white brined cheese containing microencapsulated probiotic with resistant starch were higher than that of free cells during refrigeration storage [7].

### 3.3. Viability of Microencapsulated Bifidobacterium Longum in Pineapple Juice

Effects of simulated gastric and intestinal juices and refrigeration storage on survival of microencapsulated *B. longum* with extracts and free cells is shown in Table 3. The beads were kept in pineapple juice at 4 °C for 0, 15, 30, and 45 days. The number of viable cells decreased after exposure to the simulated juices and refrigeration storage. The survival of co-encapsulated cells after exposure to the adverse conditions was better than that of non-encapsulated cells at day 15, 30, and 45 (*p <* 0.05). The highest number of *B. longum* resulted from microencapsulation with the oligosaccharides extract. The survival of *B. longum* microencapsulation with the oligosaccharides extract was reduced from 8.23 to 7.25 log_10_ CFU g^−^^1^ in the presence of the simulated juices, and that of refrigeration storage was 8.28 log_10_ CFU g^−^^1^ at day 45. Mokarram *et al.* [18] have observed that the microencapsulated probiotics with two layers of alginate resulted in a reduction of pore size which provided more favorable anaerobic environment for the bacterial cells. In a similar way from the attained result of the present study, it is envisaged that *E. americana* may have similar effects on *B. longum* cells. However, no survival of non-encapsulated cells was noted at day 30 and 45. Shabala *et al.* [23] suggested that acids reduce viability of bacteria by acidifying cytoplasm and inhibition of enzymatic reactions. Probiotic viability in fruit juice is affected by strain, culture preparation, state of the cell inoculated, oxygen level, storage temperature, and presence of prebiotics. Microencapsulation of probiotics with inulin has previously been tested for improving viability of the cells in simulated gastric and intestinal juices [5]. Rodriguez *et al.* [24] reported that microencapsulated *Lactobacillus paracasei* L26 in alginate with or without double coating resulted in good viability in both orange and peach juices after 50 days of storage at 5 °C. In addition, microencapsulated probiotics with chitosan [8] and pectin [25] were able to maintain their viability in fruit juices during refrigeration storage.

### 3.4. Sensory Analysis of Pineapple Juice

Sensory quality of pineapple juice containing microencapsulated *B. longum* with co-encapsulating agents, free cells, and control at 0, 15, 30, and 45 days of refrigeration storage are given in Table 4. Sensory scores of pineapple juice supplemented with free cells and control reduced after refrigeration storage. Overall acceptability of pineapple juice containing co-encapsulated cells was better than that of free cells at day 30 and 45 (*p <* 0.05). However, the panelist found clear no differences between day 30 and 45 pineapple juice in terms of colour properties (*p >* 0.05). The mean scores for the quality attributes of pineapple juice containing microencapsulated *B. longum* were in the range of 6.34–6.51 (where 6 = like slightly). Total evaluations in term of appearance, color, texture, and taste of the samples containing microencapsulated *B. longum* were acceptable and did not have any marked off-flavor during the storage period. Moreover, the addition of microcapsules in pineapple juice may increase gritty mouthfeel. However, pineapple juice containing free cells had marked off-flavor due to high concentration of acetic and lactic acids at day 30 and 45. Addition of microencapsulated probiotics in orange juice [26] and bread [27] improved the sensory scores than that of free cells during refrigeration storage*.*

### 3.5. Determination of Organic Acids in Pineapple Juice

**Figure 1:**
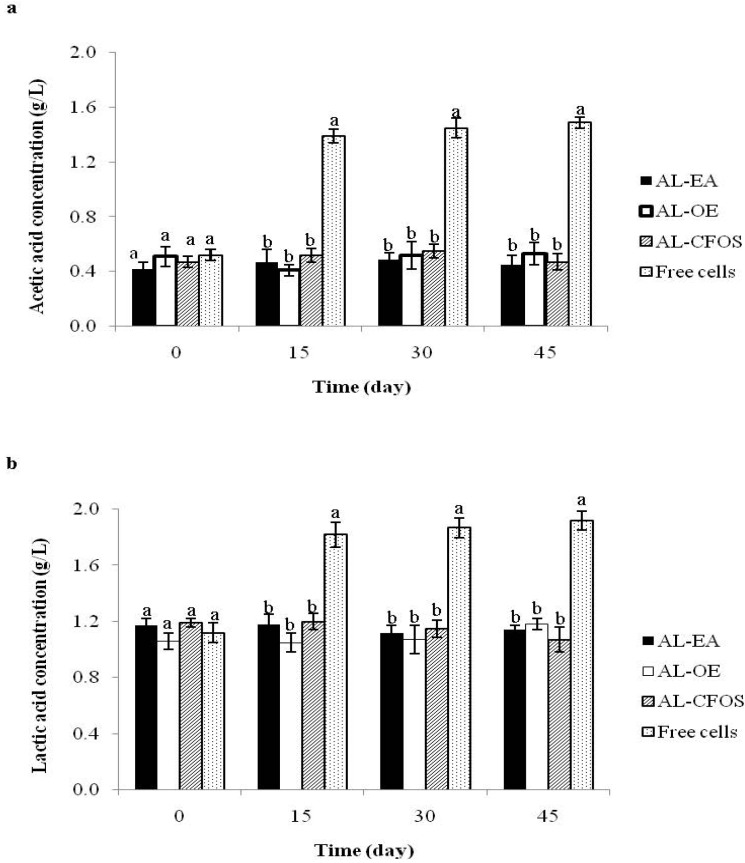
Acetic acid (**a**) and lactic acid (**b**) production of pineapple juice containing microencapsulated *Bifidobacterium longum* with *Eleutherine americana* during refrigeration storage. Alginate-*E.americana* extract (AL-EA; black bars), alginate-oligosaccharides extract (AL-OE; white bars), alginate-commercial fructo-oligosaccharides (AL-CFOS; bar with diagonal stripes), and free cells (white bar with black dots). The means ± standard deviation for at least duplicate are illustrated. Different lowercase letters in co-encapsulating agents are significantly different (*p* < 0.05).

The metabolic activity of microencapsulated *B. longum* with the extracts and free cells in pineapple juice was estimated by the production of acetic and lactic acids (Figure 1). Concentrations of acetic and lactic acids in pineapple juice were 0.48 and 1.10 g L^−^^1^, respectively (data not shown). The beads were stored in pineapple juice at 4 °C for 0, 15, 30, and 45 days. Initially, concentrations of the acetic and lactic acids were in the range of 0.42–0.52 g L^−^^1^ and 1.06–1.19 g L^−^^1^, respectively. The level of the acids in the juice containing non-encapsulated cells was higher than that of co-encapsulated cells at day 15, 30, and 45 (*p <* 0.05). The amount of the acids in the pineapple juice containing microencapsulated *B. longum* remained constant after refrigeration storage. The concentration of acetic (Figure 1a) and lactic acids (Figure 1b) in the juice containing microencapsulated *B. longum* was in the range of 0.41–0.55 and 1.05–1.20 g L^−^^1^, respectively, during refrigeration storage. Microencapsulation could reduce the problem of over acidification and thus could maintain probiotics during refrigeration storage. In addition, it is possible that local pH within the microcapsule may protect the probiotics against the external environment [28]. The type of co-encapsulating agents had no influence on concentration of the acids. The pH of the pineapple juice remained unchanged during 45 days of refrigeration storage due to high buffering capacity of the juice (data not shown). The main carbohydrates in pineapple juice are simple sugars including sucrose, glucose, and fructose. Citric acid is usually present in pineapple juice, followed by lactic acid and a small amount of acetic acid [29]. There is very little information regarding the role of acetic and lactic acids in the survival of probiotics including refrigeration storage. The acids play an important role in anti-inflammatory activity and other intestinal disorders. Probiotics can ferment the sugars into organic acids including acetic and lactic acids. Acids may increase during storage due to enzymes which are still active at 0–5 °C. Acidification could also be due to enzyme releasing from dead bacteria and hydrolyzing sugar in fruit juice [30]. Accordingly, it has previously been reported that microencapsulated probiotics had significantly lower acidity than orange and apple juices [31] and fruit food products [26] containing free cells during refrigeration storage.

**Table 1:** Survival of microencapsulated *Bifidobacterium longum* with *Eleutherine americana* in fresh milk tofu during sequential incubation in simulated human gastric and intestinal juices and refrigeration storage.

Microencapsulation	Time	Plate Count (log_10_ CFU g^−^^1^)			
	(Day)	Pre-Gastric Juice	Post-Gastric Juice	Post-Intestinal Juice	Refrigeration Storage
Co-encapsulated cells: alginate-*E. americana* extract	0	9.46 ± 0.07 ^aA^	9.40 ± 0.10 ^aA^	9.54 ± 0.19 ^aA^	9.44 ± 0.13 ^a^
Co-encapsulated cells: alginate-oligosaccharides extract		9.38 ± 0.09 ^aA^	9.32 ± 0.11 ^aA^	9.46 ± 0.04 ^aA^	9.54 ± 0.10 ^a^
Co-encapsulated cells: alginate-commercial fructo-oligosaccharides		9.30 ± 0.10 ^aA^	9.51 ± 0.05 ^aA^	9.57 ± 0.14 ^aA^	9.46 ± 0.06 ^a^
Non-encapsulated cells: free cells		9.42 ± 0.07 ^aA^	9.42 ± 0.03 ^aA^	9.34 ± 0.12 ^aA^	9.45 ± 0.14 ^a^
Co-encapsulated cells: alginate-*E. americana* extract	2	9.36 ± 0.12 ^aA^	9.45 ± 0.11 ^aA^	9.42 ± 0.06 ^aA^	9.35 ± 0.04 ^a^
Co-encapsulated cells: alginate-oligosaccharides extract		9.24 ± 0.06 ^aA^	9.32 ± 0.09 ^aA^	9.36 ± 0.03 ^aA^	9.41 ± 0.06 ^a^
Co-encapsulated cells: alginate-commercial fructo-oligosaccharides		9.16 ± 0.12 ^aA^	9.14 ± 0.04 ^aA^	9.15 ± 0.19 ^aA^	9.33 ± 0.14 ^a^
Non-encapsulated cells: free cells		8.47 ± 0.06 ^bA^	7.45 ± 0.15 ^cB^	7.30 ± 0.21 ^cB^	8.30 ± 0.10 ^b^
Co-encapsulated cells: alginate-*E. americana* extract	4	9.25 ± 0.05 ^aA^	8.37 ± 0.11 ^bB^	8.28 ± 0.09 ^bB^	9.34 ± 0.07 ^a^
Co-encapsulated cells: alginate-oligosaccharides extract		9.17 ± 0.12 ^aA^	8.15 ± 0.20 ^bB^	8.15 ± 0.10 ^bB^	9.25 ± 0.11 ^a^
Co-encapsulated cells: alginate-commercial fructo-oligosaccharides		9.25 ± 0.16 ^aA^	8.21 ± 0.14 ^bB^	8.21 ± 0.11 ^bB^	9.29 ± 0.11 ^a^
Non-encapsulated cells: free cells		8.35 ± 0.04 ^bA^	6.43 ± 0.00 ^dB^	6.46 ± 0.07 ^dB^	8.12 ± 0.15 ^b^
Co-encapsulated cells: alginate-*E. americana* extract	6	8.38 ± 0.05 ^bA^	7.36 ± 0.20 ^cB^	7.24 ± 0.16 ^cB^	8.36 ± 0.06 ^b^
Co-encapsulated cells: alginate-oligosaccharides extract		9.24 ± 0.10 ^aA^	8.52 ± 0.13 ^bB^	8.23 ± 0.07 ^bB^	9.24 ± 0.12 ^a^
Co-encapsulated cells: alginate-commercial fructo-oligosaccharides		8.45 ± 0.11 ^bA^	7.19 ± 0.15 ^cB^	7.26 ± 0.12 ^cB^	8.25 ± 0.11 ^b^
Non-encapsulated cells: free cells		6.42 ± 0.08 ^cA^	5.34 ± 0.06 ^eB^	3.56 ± 0.16 ^eC^	6.24 ± 0.06 ^c^

Values are means ± standard deviation from duplicate determinations; Different superscript uppercase letters (A,B) in the same row are significantly different among gastrointestinal conditions (*p <* 0.05); Different superscript lowercase letters (a–e) in the same column are significantly different among co-encapsulating agents (*p <* 0.05).

**Table 2:** Sensory scores of fresh milk tofu supplemented with microencapsulated *Bifidobacterium longum* with *Eleutherine americana* during refrigeration storage.

Microencapsulation	Time	Attributes				
	(Day)	Appearance	Colour	Texture	Taste	Overall Acceptability
Co-encapsulated cells: alginate-*E. americana* extract	0	7.15 ± 0.09 ^a^	7.40 ± 0.16 ^a^	7.47 ± 0.12 ^a^	7.45 ± 0.10 ^a^	7.42 ± 0.06 ^a^
Co-encapsulated cells: alginate-oligosaccharides extract		7.10 ± 0.12 ^a^	7.35 ± 0.11 ^a^	7.55 ± 0.10 ^a^	7.49 ± 0.24 ^a^	7.34 ± 0.13 ^a^
Co-encapsulated cells: alginate-commercial fructo-oligosaccharides		7.27 ± 0.07 ^a^	7.29 ± 0.08 ^a^	7.30 ± 0.09 ^a^	7.53 ± 0.14 ^a^	7.56 ± 0.09 ^a^
Non-encapsulated cells: free cells		6.95 ± 0.07 ^a^	7.48 ± 0.05 ^a^	7.37 ± 0.18 ^a^	7.40 ± 0.15 ^a^	7.45 ± 0.10 ^a^
Control		7.31 ± 0.07 ^a^	7.25 ± 0.04 ^a^	7.16 ± 0.11 ^a^	7.33 ± 0.14 ^a^	7.48 ± 0.10 ^a^
Co-encapsulated cells: alginate-*E. americana* extract	2	7.47 ± 0.16 ^a^	7.21 ± 0.20 ^a^	7.45 ± 0.17 ^a^	7.51 ± 0.13 ^a^	7.42 ± 0.14 ^a^
Co-encapsulated cells: alginate-oligosaccharides extract		7.27 ± 0.05 ^a^	6.90 ± 0.08 ^a^	7.39 ± 0.03 ^a^	7.39 ± 0.16 ^a^	7.51 ± 0.13 ^a^
Co-encapsulated cells: alginate-commercial fructo-oligosaccharides		7.13 ± 0.18 ^a^	7.14 ± 0.10 ^a^	7.26 ± 0.19 ^a^	7.35 ± 0.10 ^a^	7.34 ± 0.05 ^a^
Non-encapsulated cells: free cells		7.52 ± 0.19 ^a^	7.12 ± 0.14 ^a^	7.41 ± 0.21 ^a^	7.49 ± 0.08 ^a^	7.45 ± 0.18 ^a^
Control		7.37 ± 0.09 ^a^	7.34 ± 0.12 ^a^	7.26 ± 0.07 ^a^	7.41 ± 0.11 ^a^	7.40 ± 0.06 ^a^
Co-encapsulated cells: alginate-*E. americana* extract	4	7.27 ± 0.09 ^a^	7.35 ± 0.14 ^a^	7.18 ± 0.14 ^a^	7.27 ± 0.15 ^a^	7.26 ± 0.13 ^a^
Co-encapsulated cells: alginate-oligosaccharides extract		7.24 ± 0.05 ^a^	7.17 ± 0.15 ^a^	7.20 ± 0.20 ^a^	7.19 ± 0.10 ^a^	7.34 ± 0.04 ^a^
Co-encapsulated cells: alginate-commercial fructo-oligosaccharides		7.36 ± 0.10 ^a^	7.32 ± 0.12 ^a^	7.15 ± 0.11 ^a^	7.32 ± 0.14 ^a^	7.42 ± 0.14 ^a^
Non-encapsulated cells: free cells		6.22 ± 0.14 ^b^	7.20 ± 0.10 ^a^	6.23 ± 0.16 ^b^	6.31 ± 0.09 ^b^	6.35 ± 0.16 ^b^
Control		6.32 ± 0.10 ^b^	7.25 ± 0.12 ^a^	6.34 ± 0.06 ^b^	6.35 ± 0.08 ^b^	6.37 ± 0.12 ^b^
Co-encapsulated cells: alginate-*E. americana* extract	6	7.31 ± 0.06 ^a^	7.39 ± 0.16 ^a^	7.18 ± 0.06 ^a^	7.35 ± 0.20 ^a^	7.52 ± 0.12 ^a^
Co-encapsulated cells: alginate-oligosaccharides extract		7.16 ± 0.17 ^a^	7.28 ± 0.23 ^a^	7.23 ± 0.13 ^a^	7.19 ± 0.15 ^a^	7.45 ± 0.09 ^a^
Co-encapsulated cells: alginate-commercial fructo-oligosaccharides		7.22 ± 0.09 ^a^	7.16 ± 0.14 ^a^	7.25 ± 0.11 ^a^	7.24 ± 0.11 ^a^	7.37 ± 0.10 ^a^
Non-encapsulated cells: free cells		6.13 ± 0.14 ^b^	7.23 ± 0.09 ^a^	6.14 ± 0.00 ^b^	6.10 ± 0.14 ^b^	6.41 ± 0.13 ^b^
Control		6.20 ± 0.12 ^b^	7.27 ± 0.08 ^a^	6.27 ± 0.10 ^b^	6.22 ± 0.08 ^b^	6.40 ± 0.11 ^b^

Values are means ± standard deviation from duplicate determinations; Different superscript lowercase letters (a,b) in the same column are significantly different among co-encapsulating agents (*p* < 0.05).

**Table 3:** Survival of microencapsulated *Bifidobacterium longum* with *Eleutherine americana* in pineapple juice during sequential incubation in simulated human gastric and intestinal juices and refrigeration storage.

Microencapsulation	Time	Plate Count (log_10_ CFU/g)			
	(Day)	Pre-Gastric Juice	Post-Gastric Juice	Post-Intestinal Juice	Refrigeration Storage
Co-encapsulated cells: alginate-*E. americana* extract	0	9.55 ± 0.15 ^aA^	9.45 ± 0.12 ^aA^	9.35 ± 0.08 ^aA^	9.38 ± 0.14 ^a^
Co-encapsulated cells: alginate-oligosaccharides extract		9.42 ± 0.10 ^aA^	9.32 ± 0.16 ^aA^	9.46 ± 0.17 ^aA^	9.50 ± 0.12 ^a^
Co-encapsulated cells: alginate-commercial fructo-oligosaccharides		9.30 ± 0.07 ^aA^	9.51 ± 0.09 ^aA^	9.26 ± 0.26 ^aA^	9.47 ± 0.14 ^a^
Non-encapsulated cells: free cells		9.34 ± 0.04 ^aA^	9.42 ± 0.08 ^aA^	9.37 ± 0.09 ^aA^	9.39 ± 0.16 ^a^
Co-encapsulated cells: alginate-*E. americana* extract	15	8.20 ± 0.12 ^bA^	7.41 ± 0.15 ^cB^	7.30 ± 0.08 ^cB^	8.35 ± 0.12 ^b^
Co-encapsulated cells: alginate-oligosaccharides extract		9.15 ± 0.07 ^aA^	8.34 ± 0.04 ^bB^	8.39 ± 0.13 ^bB^	9.41 ± 0.05 ^a^
Co-encapsulated cells: alginate-commercial fructo-oligosaccharides		8.12 ± 0.13 ^bA^	7.12 ± 0.06 ^cB^	7.42 ± 0.11 ^cB^	8.26 ± 0.16 ^b^
Non-encapsulated cells: free cells		3.59 ± 0.04 ^dA^	2.65 ± 0.11 ^eB^	2.32 ± 0.04 ^eB^	3.42 ± 0.10 ^d^
Co-encapsulated cells: alginate-*E. americana* extract	30	8.25 ± 0.06 ^bA^	7.35 ± 0.14 ^cB^	7.25 ± 0.06 ^cB^	8.43 ± 0.12 ^b^
Co-encapsulated cells: alginate-oligosaccharides extract		9.14 ± 0.10 ^aA^	8.24 ± 0.13 ^bB^	8.24 ± 0.07 ^bB^	9.34 ± 0.10 ^a^
Co-encapsulated cells: alginate-commercial fructo-oligosaccharides		8.21 ± 0.09 ^bA^	7.18 ± 0.04 ^cB^	7.15 ± 0.06 ^cB^	8.26 ± 0.08 ^b^
Non-encapsulated cells: free cells		0.00 ± 0.00 ^eA^	0.00 ± 0.00 ^fA^	0.00 ± 0.00 ^fA^	0.00 ± 0.00 ^e^
Co-encapsulated cells: alginate-*E. americana* extract	45	7.21 ± 0.07 ^cA^	6.37 ± 0.11 ^dB^	6.34 ± 0.06 ^dB^	7.20 ± 0.11 ^c^
Co-encapsulated cells: alginate-oligosaccharides extract		8.23 ± 0.13 ^bA^	7.24 ± 0.05 ^cB^	7.25 ± 0.09 ^cB^	8.28 ± 0.09 ^b^
Co-encapsulated cells: alginate-commercial fructo-oligosaccharides		7.32 ± 0.10 ^cA^	6.15 ± 0.11 ^dB^	6.23 ± 0.10 ^dB^	7.34 ± 0.15 ^c^
Non-encapsulated cells: free cells		0.00 ± 0.00 ^eA^	0.00 ± 0.00 ^fA^	0.00 ± 0.00 ^fA^	0.00 ± 0.00 ^e^

Values are means ± standard deviation from duplicate determinations; Different superscript uppercase letters (A,B) in the same row are significantly different among gastrointestinal conditions (*p* < 0.05); Different superscript lowercase letters (a–f) in the same column are significantly different among co-encapsulating agents (*p* < 0.05).

**Table 4:** Sensory scores of pineapple juice supplemented with microencapsulated *Bifidobacterium longum* with *Eleutherine americana* during refrigeration storage.

Microencapsulation	Time	Attributes				
	(Day)	Appearance	Colour	Texture	Taste	Overall Acceptability
Co-encapsulated cells: alginate-*E. americana* extract	0	6.53 ± 0.17 ^a^	6.37 ± 0.22 ^a^	6.42 ± 0.17 ^a^	6.37 ± 0.13 ^a^	6.46 ± 0.11 ^a^
Co-encapsulated cells: alginate-oligosaccharides extract		6.44 ± 0.11 ^a^	6.32 ± 0.12 ^a^	6.52 ± 0.13 ^a^	6.12 ± 0.11 ^a^	6.34 ± 0.15 ^a^
Co-encapsulated cells: alginate-commercial fructo-oligosaccharides		6.35 ± 0.05 ^a^	6.51 ± 0.09 ^a^	6.33 ± 0.16 ^a^	6.06 ± 0.04 ^a^	6.51 ± 0.12 ^a^
Non-encapsulated cells: free cells		6.42 ± 0.09 ^a^	6.44 ± 0.07 ^a^	6.46 ± 0.15 ^a^	6.17 ± 0.15 ^a^	6.45 ± 0.20 ^a^
Control		6.37 ± 0.09 ^a^	6.35 ± 0.12 ^a^	6.34 ± 0.10 ^a^	6.30 ± 0.11 ^a^	6.41 ± 0.15 ^a^
Co-encapsulated cells: alginate-*E. americana* extract	15	6.46 ± 0.17 ^a^	6.36 ± 0.22 ^a^	6.40 ± 0.12 ^a^	6.37 ± 0.13 ^a^	6.47± 0.14 ^a^
Co-encapsulated cells: alginate-oligosaccharides extract		6.41 ± 0.06 ^a^	6.41 ± 0.09 ^a^	6.39 ± 0.10 ^a^	6.41 ± 0.09 ^a^	6.45 ± 0.11 ^a^
Co-encapsulated cells: alginate-commercial fructo-oligosaccharides		6.36 ± 0.23 ^a^	6.35 ± 0.11 ^a^	6.29 ± 0.15 ^a^	6.35 ± 0.14 ^a^	6.37 ± 0.09 ^a^
Non-encapsulated cells: free cells		6.34 ± 0.24 ^a^	6.45 ± 0.21 ^a^	6.35 ± 0.14 ^a^	6.38 ± 0.13 ^a^	6.49 ± 0.10 ^a^
Control		6.45 ± 0.08 ^a^	6.37 ± 0.10 ^a^	6.30 ± 0.08 ^a^	6.35 ± 0.14 ^a^	6.40 ± 0.12 ^a^
Co-encapsulated cells: alginate-*E. americana* extract	30	6.61 ± 0.06 ^a^	6.34 ± 0.12 ^a^	6.40 ± 0.09 ^a^	6.25 ± 0.13 ^a^	6.46 ± 0.14 ^a^
Co-encapsulated cells: alginate-oligosaccharides extract		6.39 ± 0.15 ^a^	6.35 ± 0.15 ^a^	6.37 ± 0.15 ^a^	6.18 ± 0.15 ^a^	6.38 ± 0.12 ^a^
Co-encapsulated cells: alginate-commercial fructo-oligosaccharides		6.42 ± 0.14 ^a^	6.38 ± 0.14 ^a^	6.25 ± 0.11 ^a^	6.26 ± 0.12 ^a^	6.42 ± 0.20 ^a^
Non-encapsulated cells: free cells		5.52 ± 0.13 ^b^	6.40 ± 0.07 ^a^	5.46 ± 0.09 ^b^	5.31 ± 0.12 ^b^	5.51 ± 0.13 ^b^
Control		5.40 ± 0.09 ^b^	6.34 ± 0.10 ^a^	5.37 ± 0.08 ^b^	6.34 ± 0.12 ^a^	5.54 ± 0.10 ^b^
Co-encapsulated cells: alginate-*E. americana* extract	45	6.29 ± 0.06 ^a^	6.24 ± 0.15 ^a^	6.34 ± 0.10 ^a^	6.22 ± 0.10 ^a^	6.40 ± 0.13 ^a^
Co-encapsulated cells: alginate-oligosaccharides extract		6.31 ± 0.15 ^a^	6.17 ± 0.20 ^a^	6.41 ± 0.12 ^a^	6.18 ± 0.08 ^a^	6.36 ± 0.11 ^a^
Co-encapsulated cells: alginate-commercial fructo-oligosaccharides		6.25 ± 0.14 ^a^	6.19 ± 0.14 ^a^	6.39 ± 0.13 ^a^	6.26 ± 0.11 ^a^	6.42 ± 0.09 ^a^
Non-encapsulated cells: free cells		5.35 ± 0.00 ^b^	6.12 ± 0.00 ^a^	5.34 ± 0.00 ^b^	5.31 ± 0.16 ^b^	5.37 ± 0.15 ^b^
Control		5.32 ± 0.12 ^b^	6.37 ± 0.10 ^a^	5.30 ± 0.09 ^b^	6.32 ± 0.09 ^a^	5.57 ± 0.15 ^b^

Values are means ± standard deviation from duplicate determinations; Different superscript lowercase letters (a,b) in the same column are significantly different among co-encapsulating agents (*p <* 0.05).

## 4. Conclusions

Microencapsulated *Bifidobacterium longum* with *Eleutherine americana* extract and its oligosaccharides extract in fresh milk tofu and pineapple juice showed better survival than free cells after sequential incubation in simulated gastric and intestinal juices and refrigeration storage. Microencapsulated cells appeared to be effective in improving the sensory quality of the food products. Addition of microencapsulated cells in the pineapple juice caused a slowing of post-acidification during refrigeration storage. Further studies are warranted in animal models before potential application for consumers.
